# Effects of entecavir and tenofovir disoproxil fumarate on the incidence and severity of COVID-19 in patients with chronic hepatitis B

**DOI:** 10.1186/s12879-023-08838-0

**Published:** 2023-11-30

**Authors:** Xingmei Liao, Yujie Fan, Chunxiu Zhong, Siru Zhao, Liangxu Guo, Wenjuan Tan, Junhua Yin, Rong Fan

**Affiliations:** 1 Department of Infectious Diseases, Nanfang Hospital, Southern Medical University, Guangzhou, China; 2 Guangdong Provincial Key Laboratory of Viral Hepatitis Research, Nanfang Hospital, Southern Medical University, Guangzhou, China; 3 Guangdong Provincial Clinical Research Center for Viral Hepatitis, Nanfang Hospital, Southern Medical University, Guangzhou, China; 4 Key Laboratory of Infectious Diseases Research in South China, Ministry of Education, Nanfang Hospital, Southern Medical University, Guangzhou, China

**Keywords:** Tenofovir, Entecavir, COVID-19, Chronic Hepatitis B

## Abstract

**Background:**

Whether different anti-hepatitis B virus (HBV) drugs have different effects on COVID-19 is controversial. We aimed to evaluate the incidence of COVID-19 in chronic hepatitis B (CHB) patients receiving anti-HBV treatment, and to compare the impact of entecavir (ETV) and tenofovir disoproxil fumarate (TDF) on the severity of COVID-19.

**Methods:**

CHB outpatients were enrolled from December 2022 to February 2023. Questionnaires were used to collect whether subjects were currently or previously had COVID-19 within the past 2 months, and the information of symptoms, duration, and severity if infected.

**Results:**

Six hundred thirty CHB patients were enrolled, 64.3% (405/630) patients were currently or previously had COVID-19. No COVID-19 patient required hospitalization, intensive care unit admission, oxygen support or died. Majority of patients reported mild (32.8% [133/405]) and moderate (48.1% [195/405]) symptoms. After propensity score matching, 400 matched patients were obtained (ETV: 238; TDF: 162), among which the incidences of COVID-19 were comparable between ETV and TDF-treated patients (60.1% [143/238] vs. 64.2% [104/162], *p* = 0.468). The proportion of patients complicated with any symptom caused by COVID-19 were also similar (ETV vs. TDF: 90.9% [130/143] vs. 91.3% [95/104], *p* = 1.000). In addition, the severity of overall symptom was comparable between ETV and TDF-treated patients, in terms of proportion of patients complicated with severe symptom (9.8% vs. 8.7%, *p* = 0.989), symptom duration (4.3 vs. 4.3 days, *p* = 0.927), and symptom severity score (4.1 vs. 4.0, *p* = 0.758). Subgroup analysis supported these results.

**Conclusions:**

During the current pandemic, the vast majority of CHB patients experienced non-severe COVID-19, and ETV and TDF did not affect COVID-19 severity differently.

## Introduction

By March 2023, more than 676 million people have been infected with SARS-CoV-2 and 6.8 million have been declared dead from COVID-19 [1]. SARS-CoV-2 has undergone several mutations. Currently, various strains of the global epidemic all belong to the sub branch of the omicron variant. Although the global epidemic has gradually under control with the development and application of various vaccines and antiviral drugs, the infection situation and severity of people with underlying diseases still need to be concerned [2]. From early December 2022, a huge number of patients were infected with SARS-CoV-2 in a short period of time in China, which allowed us to observe the incidence and severity of COVID-19 in chronic hepatitis B (CHB).

Recently, several studies reported that, tenofovir disoproxil fumarate (TDF), as the first-line anti- hepatitis B virus (HBV) drug, could reduce the risk of severe COVID-19 [3], and may protect against COVID-19-related events such as hospitalization and intensive care unit (ICU) admission [4–9]. In vitro studies also demonstrated that tenofovir partly inhibits the SARS-CoV-2 RNA-dependent RNA-polymerase (RdRp) [10–12], and triphosphate forms of tenofovir are believed to be incorporated by SARS-CoV-2 RdRp and retard polymerase extension [13]. In addition, TDF plus emtricitabine appeared to accelerate the natural clearance of nasopharyngeal SARS-CoV-2 viral burden [14]. These could explain why the TDF could inhibit SARS-CoV-2. However, there were also studies suggest that anti-HBV agents including TDF and entecavir (ETV) have no beneficial effect to COVID-19 in CHB patients and general population [15, 16]. An in vitro study showed that TDF was inactive against SARS-CoV-2, and this result was confirmed by the lack of interaction between the respective NRTI-triphosphates and SARS-CoV-2 RdRp observed both in biochemical assays and in structural modelling analyses [17]. Therefore, whether TDF could reduce the risk of severe COVID-19 is controversial, which needs further investigation.

In this study, we aimed to evaluate the incidence of COVID-19 in CHB patients receiving anti-HBV treatment, and to compare the impact of ETV and TDF on the severity of COVID-19.

## Methods

### Study population

​Between December 19, 2022 to February 17, 2023, a total of 630 outpatients with CHB were enrolled from Nanfang Hospital, Southern Medical University. Patients were recruited if they were above 18 years, diagnosed as CHB, treated with TDF or ETV, and willing to provide personal information. Exclusion criteria were patients with human immunodeficiency virus (HIV) or other chronic liver diseases, including other virus hepatitis, alcoholic liver diseases, and drug-induced liver injury. The study was approved by the Medical Ethics Committee of Nanfang Hospital, Southern Medical University. All patients provided written informed consent to participate.

### Data collection

Detailed demographic information was collected at enrolment, including age, sex, height, weight, use of alcohol and tobacco, vaccination status, cirrhosis status and complications such as hypertension, diabetes and hyperlipidemia.

By using a survey questionnaire, we collected whether the subjects were currently or previously diagnosed as COVID-19 within the past 2 months, and the information of clinical symptoms, duration, and severity were collected for COVID-19 patients. The symptoms collected included overall symptom and 12 specific symptoms. Overall symptom is the general assessment of the discomfort caused by COVID-19. Twelve specific symptoms include fever, nasal obstruction, sore throat, dyspnea, cough, muscular soreness, headache, chill, anosmia, nausea, vomiting, and diarrhea.

The Visual Analog Score (VAS) [18, 19] was used to assess the severity of COVID-19 related clinical symptoms. VAS usually uses a 10 cm long straight line, and patients mark on it according to the degree of discomfort they feel. The higher the score, the stronger the discomfort. In our study, score 0 was considered asymptomatic or negligible. Scores 1–3 were considered mild and self-relieving. Scores 4–6 were considered moderate and can be relieved with medication. Scores 7–10 were considered severe and requires outpatient medical attention.

### Statistical analysis

Categorical variables were expressed as counts and percentages, and continuous variables were reported as means ± standard deviations or quartiles. Group comparisons were conducted using the Pearson’s chi-square test or the Fisher’s exact test for categorical parameters, and the student’s t test or the Mann–Whitney U test for continuous parameters. Propensity score matching (PSM) was performed to balance the confounding factors among different groups, such as sex, age, complication and cirrhosis. We did PSM in a ratio 1:2 using nearest-neighbor algorithms with a caliper width of 0.2 of the pooled standard deviations. Two-sided *p* values < 0.05 were considered statistically significant. All statistical analyses were performed with R Statistical Software version 4.2.1 and SPSS Statistics package version 26.0.

## Results

### Clinical characteristics of all patients

A total of 630 CHB patients were enrolled in the analysis. Table 1 presented the clinical characteristic of all patients. The average age was 45.4 ± 9.0 years, with 81.9% (516/630) of males and 47.9% (302/630) of cirrhosis. 92.5% (583/630) of patients completed at least 1 dose of SARS-CoV-2 vaccine. Among the overall patients, 66.0% (416/630) and 34.0% (214/630) were receiving ETV and TDF treatment, respectively.

**Table 1:** Clinical Characteristics of all patients

	**Total (*N* = 630)**	**COVID-19 (*N* = 405)**	**Non-COVID-19 (*N* = 225)**	***p* value**
Male, n (%)	516 (81.9)	326 (80.5)	190 (84.4)	0.260
Age, years	45.4 ± 9.0	43.9 ± 8.4	48.1 ± 9.3	< 0.001
Age group, n (%)				< 0.001
[18,45)	319 (50.6)	230 (56.8)	89 (39.6)	
≥ 45	311 (49.4)	175 (43.2)	136 (60.4)	
BMI (kg/m^2^)	23.2 ± 3.1	23.3 ± 3.3	23.0 ± 2.7	0.206
Smoking, n (%)	102 (16.2)	57 (14.1)	45 (20.0)	0.068
Drinking, n (%)	34 (5.4)	24 (5.9)	10 (4.4)	0.545
Cirrhosis, n (%)	302 (47.9)	177 (43.7)	125 (55.6)	0.006
Liver cancer, n (%)	17 (2.7%)	9 (2.2%)	8 (3.6%)	0.464
Treatment, n (%)				0.117
ETV	416 (66.0)	158 (70.2)	258 (63.7)	
TDF	214 (34.0)	67 (29.8)	147 (36.3)	
Hypertension, n (%)	54 (8.6)	28 (6.9)	26 (11.6)	0.065
Diabetes, n (%)	24 (3.8)	8 (2.0)	16 (7.1)	0.003
Hyperlipemia, n (%)	58 (9.2)	37 (9.1)	21 (9.3)	1.000
Vaccination^a^, n (%)	583 (92.5)	374 (92.3)	209 (92.9)	0.928

*ETV* Entecavir, *TDF* Tenofovir disoproxil fumarate

*p* value, assessed by χ2 or t test

^a^get vaccinated against SARS-CoV-2

### Incidence and clinical characteristics of COVID-19 patients

As of the date of questionnaire collection, 64.3% (405/630) patients were currently or previously diagnosed as COVID-19. Among the 405 COVID-19 patients, the proportion of patients complicated with any symptom caused by COVID-19 was 89.9% (364/405), with average duration of 4.3 days. The percentage of self-reported mild and moderate symptoms were 33.1% (134/405) and 48.4% (196/405), respectively. Only 8.4% (34/405) reported severe symptoms. Notably, no one required hospitalization, ICU admission, oxygen support or died due to COVID-19. The most common symptom was fever (304/405, 75.1%), followed by cough (257/405, 63.5%), sore throat (213/405, 52.6%), muscular soreness (205/405, 50.6%), headache (199/405, 49.1%), nasal obstruction (195/405, 48.1%) and chill (190/405, 46.9%). None of the 12 specific symptoms lasted more than 4 days on average. Compared with non-COVID-19 patients, COVID-19 patients were more likely to be younger (43.9 vs. 48.1 years, *p* < 0.001) and had lower rates of cirrhosis (43.7% vs. 55.6%, *p* = 0.006) and diabetes (2.0% vs. 7.1%, *p* = 0.003). The other clinical characteristics between the patients with or without COVID-19 were comparable (Table 1).

### Comparison of incidence and severity of COVID-19 between ETV and TDF-treated patients

**Fig. 1:**
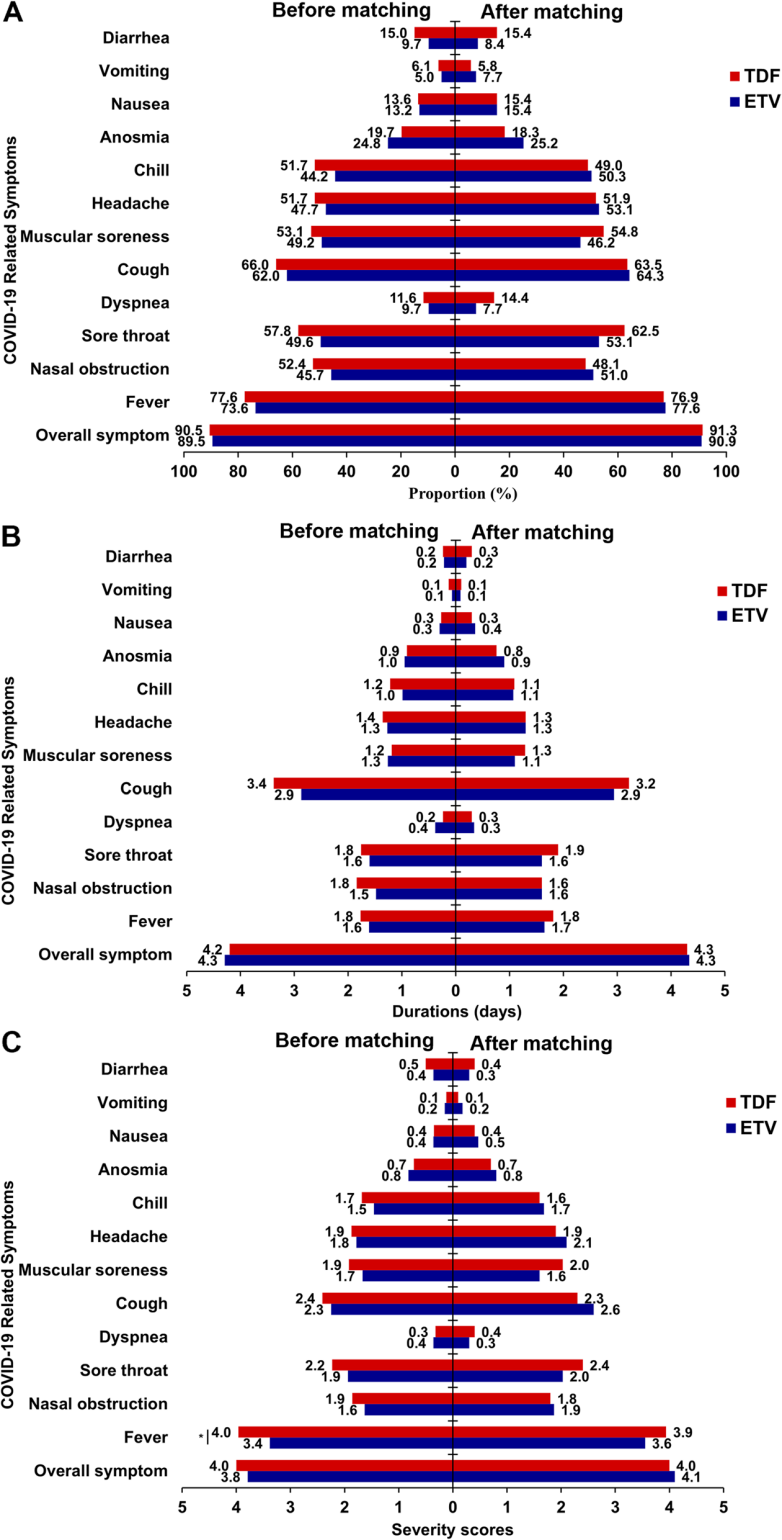
COVID-19 related symptoms in patients receiving ETV and TDF treatment before and after matching. The proportion of patients complicated with symptoms caused by COVID-19 (**A**), symptom duration (**B**) and symptom severity score (**C**) ETV, Entecavir. TDF, Tenofovir disoproxil fumarate. *, *p* < 0.05

**Fig. 2:**
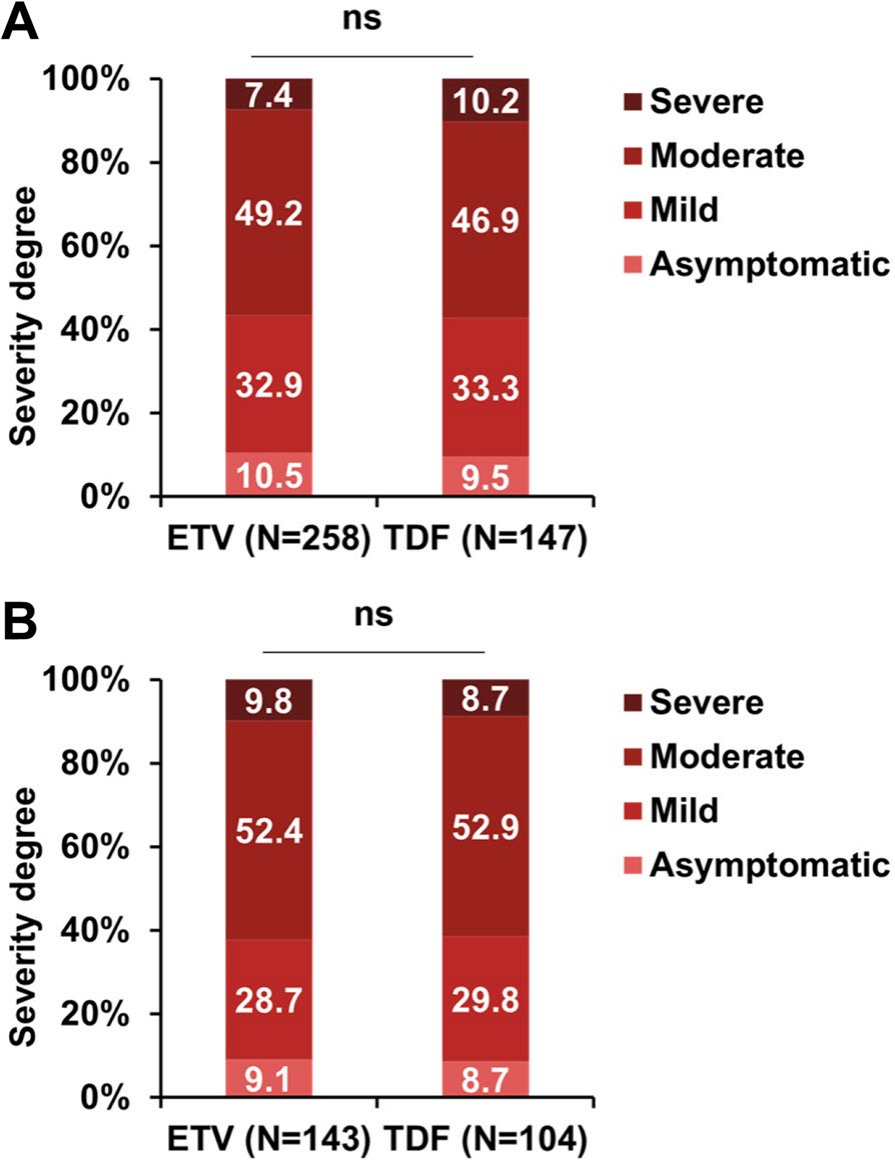
Comparison between patients receiving ETV and TDF treatment in terms of the distribution of severity degree of overall symptom caused by COVID-19 before (**A**) and after (**B**) matching. ETV, Entecavir. TDF, Tenofovir disoproxil fumarate. Ns: no significance

Among the TDF-treated patients, the incidence of COVID-19 was 68.7%, which was comparable with ETV-treated patients (62.0%, *p* = 0.117) (Table 2). Besides, the proportion of patients complicated with any symptom caused by COVID-19 (89.5% vs. 90.5%, *p* = 0.896), symptom duration (4.3 vs. 4.2 days, *p* = 0.753), symptom severity score (3.8 vs. 4.0,* p* = 0.548) and symptom severity degree (mild and moderate: 82.1% vs. 80.2%, *p* = 0.780) had no significant difference between the two groups (Figs. 1, and 2A). ​

**Table 2:** Clinical characteristics of ETV and TDF treated patients before and after propensity score matching

	**Before matching**			**After matching**		
	**ETV (*N* = 416)**	**TDF (*N* = 214)**	***p* value**	**ETV (*N* = 238)**	**TDF (*N* = 162)**	***p* value**
Male, n (%)	352 (84.6)	164 (76.6)	0.019	205 (86.1)	138 (85.2)	0.904
Age, years	46.7 ± 9.0	42.8 ± 8.3	< 0.001	44.7 ± 8.1	44.0 ± 8.0	0.417
Age group, n (%)			< 0.001			0.617
< 45 years	188 (45.2)	131 (61.2)		122 (51.3)	88 (54.3)	
≥ 45 years	228 (54.8)	83 (38.8)		116 (48.7)	74 (45.7)	
BMI (kg/m^2^)	23.3 ± 3.0	22.9 ± 3.2	0.106	23.2 ± 3.1	23.1 ± 3.1	0.736
Smoking, n (%)	70 (16.8)	32 (15.0)	0.624	41 (17.2)	27 (16.7)	0.991
Drinking, n (%)	24 (5.8)	10 (4.7)	0.696	11 (4.6)	10 (6.2)	0.650
Cirrhosis, n (%)	218 (52.4)	84 (39.3)	0.002	110 (46.2)	66 (40.7)	0.327
Liver cancer, n (%)	13 (3.1%)	4 (1.9%)	0.508	9 (3.8%)	4 (2.5%)	0.660
Hypertension, n (%)	41 (9.9)	13 (6.1)	0.146	9 (3.8)	8 (4.9)	0.756
Diabetes, n (%)	20 (4.8)	4 (1.9)	0.108	5 (2.1)	1 (0.6)	0.436
Hyperlipemia, n (%)	39 (9.4)	19 (8.9)	0.953	15 (6.3)	13 (8.0)	0.643
Vaccination^a^, n (%)	379 (91.1)	204 (95.3)	0.080	217 (91.2)	157 (96.9)	0.038
COVID-19, n (%)	258(62.0)	147(68.7)	0.117	143 (60.1)	104 (64.2)	0.468

*ETV* Entecavir, *TDF* Tenofovir disoproxil fumarate, *CHB* Chronic Hepatitis B

*p* value, assessed by χ2 or t test

^a^get vaccinated against SARS-CoV-2

Compared with TDF-treated patients, ETV-treated patients were more often to be male (84.6% vs. 76.6%, *p* = 0.019), older (46.7 vs. 42.8 years, *p* < 0.001), and had a higher rate of cirrhosis (52.4% vs. 39.3%, *p* = 0.002) (Table 2). Hence, we conducted PSM to balance these confounding factors. After PSM, 400 matched patients were obtained, of which 238 on ETV and 162 on TDF (Table 2). The incidences of COVID-19 between patients receiving ETV and TDF remains similar (60.1% [143/238] vs. 64.2% [104/162], *p* = 0.468). The proportions of patients complicated with any symptom caused by COVID-19 were also similar (ETV vs. TDF: 90.9% [130/143] vs. 91.3% [95/104], *p* = 1.000). In addition, the severity of overall symptom among the ETV patients was comparable with that among TDF treated patients, in terms of proportion of patients complicated with severe symptom (9.8% vs. 8.7%, *p* = 0.989), symptom duration (4.3 vs. 4.3 days, *p* = 0.927), and symptom severity score (4.1 vs. 4.0, *p* = 0.758) (Figs. 1, and 2B).

As for the specific 12 COVID-19 symptoms, only the severity score of fever (4.0 vs, 3.4, *p* = 0.035) showed differences between groups before PSM (Fig. 1). After PSM, the proportion of patients complicated with any symptom caused by COVID-19, symptom duration, or symptom severity score of all 12 symptoms in the ETV-treated group were similar to those in TDF-treated group (Fig. 1).

### Subgroup analysis

**Fig. 3:**
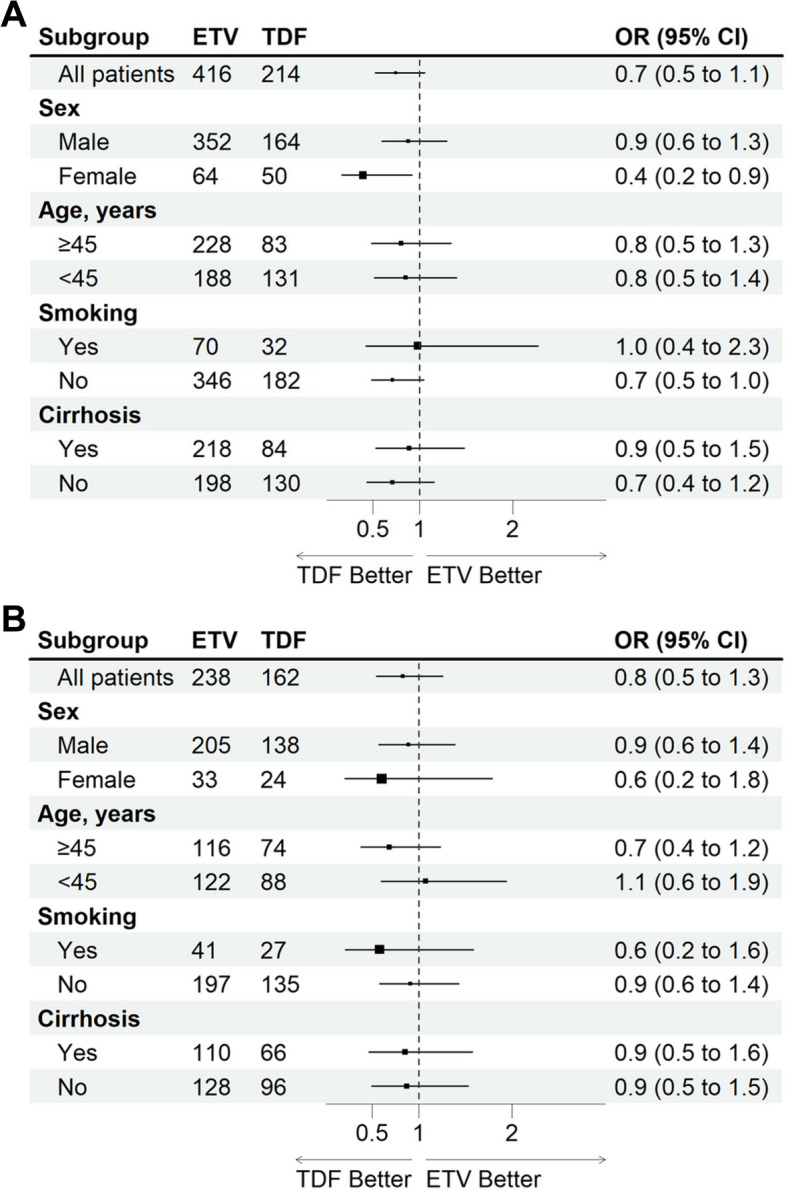
The impact of ETV and TDF on the occurrence of COVID-19 in different subgroups (**A**) before and (**B**) after matching. p-value was assessed by logistic regression analysis. ETV, Entecavir. TDF, Tenofovir disoproxil fumarate. OR, odds ratio; CI, confidence interval

**Fig. 4:**
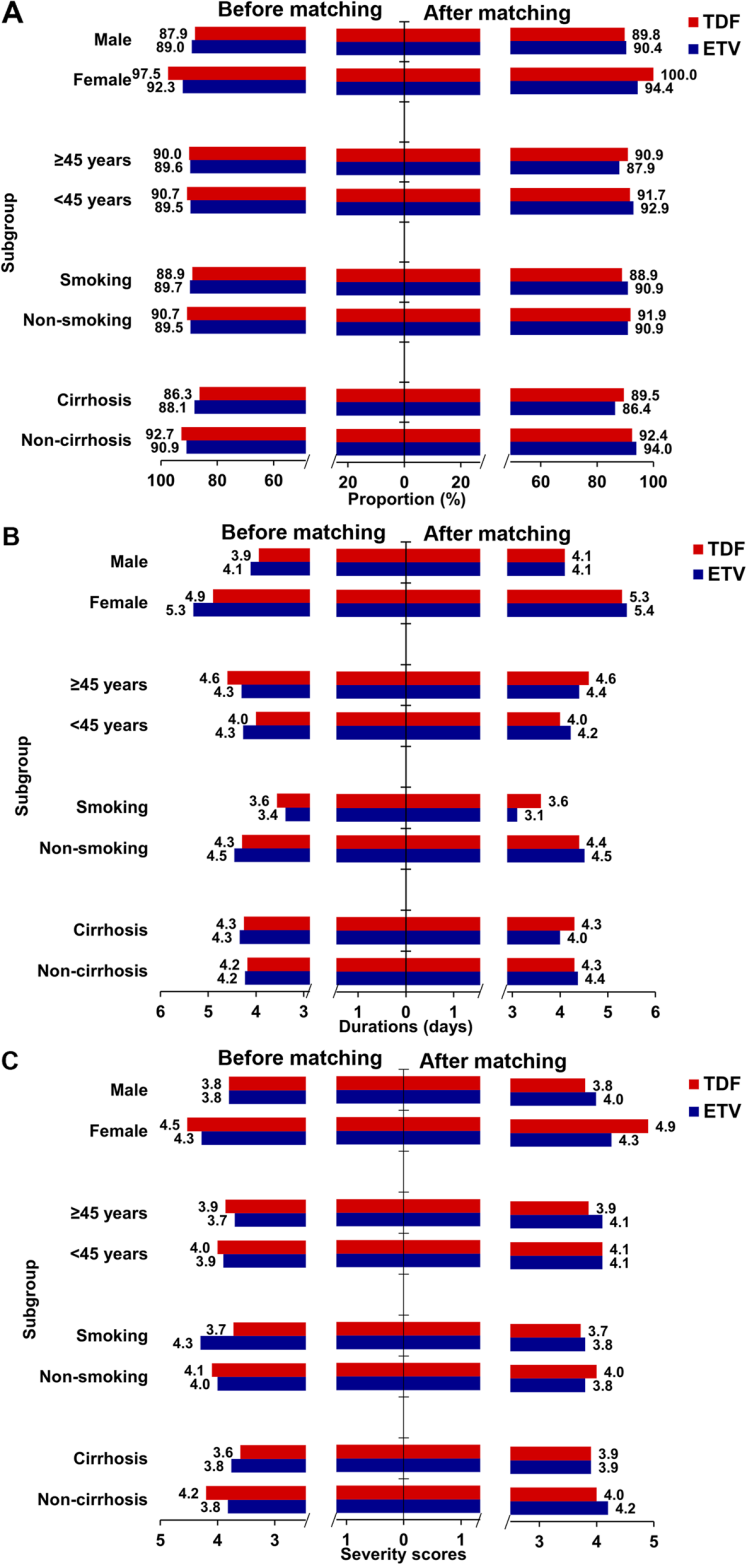
The proportion of patients complicated with overall symptom caused by COVID-19 (**A**), symptom duration (**B**) and symptom severity score (**C**) in different subgroups before and after matching (all *p* > 0.05). ETV, Entecavir. TDF, Tenofovir disoproxil fumarate

We also conducted subgroup analysis according to sex, age, smoking and cirrhosis status. The results showed that there was no difference in the effect of using ETV or TDF on the incidence of COVID-19 in these subgroups. Although in women, the use of TDF was an independent predictor of COVID-19’s occurrence (Fig. 3A). However, this difference disappeared in the matched population (Fig. 3B). In these subgroups, the differences in the proportion of patients complicated with overall symptom, symptom duration, and symptom severity score were all comparable between the ETV-treated and TDF-treated groups (Fig. 4).

## Discussion

In this study, we investigated the incidence, duration, and severity of COVID-19 among 630 CHB patients receiving ETV or TDF. Our results showed that ETV or TDF treatment had similar impacts on the incidence, duration, and severity of COVID-19 in CHB patients. After adjusting for multiple confounding factors, the conclusions remained consistent.

In the current study, 64.3% (405/630) of CHB patients had COVID-19. The vast majority of COVID-19 patients self-reported mild and moderate symptoms, and only 8.4% (34/405) reported severe symptoms. Notably, no one required hospitalization, ICU admission, oxygen support or died due to COVID-19. This was much different with another study conducted in Spanish CHB, where the incidence of COVID-19 was 2.5%, 39.3% need hospitalization, 18.8% presented severe COVID-19, and 4.3% of them required ICU admission. 10.3% (12/117) received ventilatory support and 5.1% (6/117) died [3]. This discrepancy may due to differences in the study population, study period and epidemic strains. With the continuous mutation of SARS-CoV-2 virus, omicron variant strains were widely prevalent, and the lethality of the omicron variant was significantly reduced. More and more infected people were presenting as non-severe cases. Previous studies reported that, patients infected with the omicron variant had a significantly lower risk of hospitalization (0.2%—4.1%), admission to the ICU (0.1%—0.5%) and death (0.46%), compared with the delta variant [20–24]. In the current study, we evaluated the incidence, duration, and severity of COVID-19 among CHB population in China during the omicron epidemic, while the other study investigated those indexes of COVID-19 in the first year of the COVID-19 epidemic. This could explain the inconsistence of incidence rate and severity of COVID-19 between the two studies.

Moreover, it should be mentioned that although the proportion of severe COVID-19 patients was lower, we found that the rate of symptomatic COVID-19 was still high during the omicron epidemic. In our study, the rate of patients experienced with at least one COVID-19 related symptom was about 90%, and the corresponding rate for each symptom ranged from 5.0% to 77.6%. Previous studies also reported that the rate of each symptom (i.e., nasal obstruction, headache, sore throat and cough, etc.) for patients who tested positive for SARS-CoV-2 ranged from 4.3%-76.5% when omicron was dominant, which was similar to our results [21].

Besides, the incidence of COVID-19 was comparable between ETV-treated patients and TDF-treated patients in this study. This result was partially supported by previous studies, which found that TDF was not related to the reduction of incidence of COVID-19 [4–9]. Although a study suggested that antiviral agents including TDF and ETV were associated with a decrease in the positive rate of SARS-CoV-2 [16]. However, it is worth noting that in the latter study, only 50 patients received antiviral drugs, and the effective sample size was small, which may lead to deviations in the results. Furthermore, our results of subgroup analysis showed that TDF was effective in reducing the incidence of COVID-19 in women before matching for the confounding factors, but after matching, this effect disappeared. This was consistent with another prospective multicenter cohort study on HIV infected people. They found that after using the adjusted Cox regression model, the potential protective effect of TDF /FTC on the incidence of COVID-19 disappeared [8]. Therefore, based on the evidences from the current study, we believed that TDF and ETV had similar impacts in the incidence of COVID-19.

Moreover, the results of our study showed that there was no significant difference in the proportion of patients complicated with symptoms caused by COVID-19, symptom duration, symptom severity score and symptom severity degree between ETV- and TDF-treated patients before and after matching. These results were consistent with previous studies. In a Korean nationwide cohort, among 7,723 SARS-CoV-2 positive patients, 480 (6.2%) patients were diagnosed as severe COVID-19 and 237 (3.1%) died during hospitalization. And 26 (5.4%) patients with severe COVID-19 and 12 (5.1%) fatalities occurred in CHB patients [16]. Researchers found that antiviral agents, including TDF and ETV, was not associated with the severe clinical outcome of COVID-19 [16]. Except that, the results from an open-label, double-randomized, phase 3 pragmatic clinical trial in 355 subjects suggested that TDF has no beneficial effect to severe COVID-19 [15]. Based on the above evidence, we considered that ETV and TDF did not affect COVID-19 severity differently in CHB patients.

Interestingly, there were also some studies that differ from our results. The reason behind it may be the difference in the severity degree distribution of COVID-19 among the subjects. The main population assessed in this study was non-severe COVID-19 patients, while most of the previous studies have focused on severe COVID-19 patients. For instance, a study of the Spanish CHB population identified 117 cases of COVID-19, of which 39.3% cases required hospitalization and 18.8% presented severe COVID-19 [3]. They found that ETV patients more often had severe COVID-19, required ICU, ventilatory support, had longer hospitalization, or died. And TDF can play a protective role in COVID-19 patients with CHB compared with ETV [3].The inverse probability of treatment weighting propensity score also showed that TDF can reduce the risk of severe COVID-19 by 6 times [3]. What’s more, several studies found that compared with other antiretroviral drugs, TDF can protect against COVID-19-related events such as hospitalization and ICU admission in HIV-positive patients [4–9, 25]. In summary, the above studies suggested that TDF is beneficial for severe COVID-19.

In silico and in vitro studies suggested that TDF inhibits the SARS-CoV-2 RNA-dependent RNA-polymerase (RdRp) [10–12], and TDF plus emtricitabine can accelerate the natural clearance of nasopharyngeal SARS-CoV-2 viral burden [14]. In addition, TDF also decreases the production of interleukins-8 and interleukins-10, both of which have been shown to reduce the severity of COVID-19 [26]. However, a comprehensive set of in vitro data indicated that tenofovir (TFV), tenofovir alafenamide (TAF), TDF, and FTC were inactive against SARS-CoV-2 [17]. None of these drugs showed any significant in vitro anti-SARS-CoV-2 effect at concentrations up to 100-fold higher than the clinically relevant levels [17]. Moreover, structural modeling further demonstrated poor fitting of these nucleoside/tide reverse transcriptase inhibitors (NRTIs) active metabolites at the SARS-CoV-2 RdRp active site [17]. Their data indicated that TDF was unlikely direct-antivirals against SARS-CoV-2 [17]. More researches were needed to confirm whether TDF benefits to COVID-19.

There are indeed some limitations in this study. Firstly, this was a single-center cross-sectional study, and the results from subgroups analysis with small sample size may not be unreliable and need to be validated in a larger sample size. Secondly, VAS was used to evaluate the severity of COVID-19, which is greatly influenced by the subjective factors of the patients and may cause some deviation. Thirdly, the biochemical and virological information were not collected in the current study, hence we were unable to evaluate the prevalence of liver or kidney injury, as well as the change of HBV-related virological markers during COVID-19 infection among CHB patients, which warrants further investigation.

In conclusion, the vast majority of CHB patients experienced non-severe COVID-19 during the current pandemic. ETV and TDF did not affect COVID-19 severity differently in CHB patients. More perfect research design is needed to further explore the impact of the two drugs on COVID-19.

## Data Availability

The data generated during and/or analyzed during the current study are available from the corresponding author on reasonable request.

## Abbreviations

HBV: Hepatitis B virus
CHB: Chronic hepatitis B
ETV: Entecavir
TDF: Tenofovir disoproxil fumarate
ICU: Intensive care unit
RdRp: RNA-dependent RNA-polymerase
HIV: Human immunodeficiency virus
VAS: Visual Analog Score
PSM: Propensity score matching
NRTIs: Nucleoside/tide reverse transcriptase inhibitors
